# Politeness to Patients

**Published:** 1919-02-15

**Authors:** 


### THE MATRONS' AND SISTERS' DEPARTMENT.

### POLITENESS TO PATIENTS.

By CADUCEUS.

A seasonable topic as times go, but it is not the present democratic trend of home politics which principally suggests it. What has brought it to mind is a little series of like events, a little tale of stimuli which in sum have caused the realisation of a certain contrast. That contrast is the one observable between the behaviour to patients of the staffs of the newer municipal medical institutions, and of those of the older voluntary hospitals. Perhaps instead of "observable" one should say "observed," for perceptions other than those of the writer are in question. The mothers at a municipal clinic the other day were loud in complaint of the manners towards them of the nurses at the neighbouring hospital. A sanatorium patient going out for an operation to a large provincial infirmary, said on his return, "Ah, it's nice to be served your breakfast comfortably! *There* it was 'Want any bread, Jack?'" Patients are by way of noticing personal experiences, like the rest of humanity.

A hospital house surgeon taking *locum tenens* duty at an institution under the National Insurance Act had to put up a weekly list of patients on the notice-board. All the men's names were given *tout court*, without any "Mr." such as had appeared on all similar lists previously. The change was remarked upon. Was it a wise one? Did the saving of a second's time justify it? Give a working-class male patient this modest handle to his name (some schoolmasters give it, partly in fun but more in courtesy, to their pupils) and see how infallibly he will call you "Sir" in his reply. Ask him to be seated, and note how he thanks you at once. By such an infinitesimal expenditure of energy you have assured him of your benevolence, won his goodwill, and improved your own manners.

Why, after all, should not that bad old hospital custom of calling every elderly male inmate "Daddy" be demolished? It is nothing but a presumption: really a vestigial relic of mid-Victorian times, when the vinous gentleman of the period would on small provocation address his valet or his waiter as "you lying hound." And to cite more material reasons for now honouring it in the breach, labour dignitaries are hard to tell by their appearance, and there are plenty of possible grounds of difference with a working-class hospital governor which would prove more defensible than a charge of lack of manners.

What is especially wanting on our part is the perception of how well off, in this still feudal land, we are in the matter of intercourse with the common people. They stand so little on their rights. How spontaneously (well, yes, it is necessary at times) one interrupts them when speaking, and how submissively they take it! How they will wait for one! Let us observe ourselves next time we are not sure of our way in the street, and note how we spare well-dressed passers-by, and pitch on one of lower social rank to trouble with our queries; who stops, giving us all his attention, and directs us, and is cut short the instant we have got the clue from him. Things may be uncomfortably different before very long. In America, for instance, instead of the civil "Tickets, please sir!" a traveller may not impossibly encounter, "Young man, where to h—'s your ticket?" In that country Mr. Henry James has remarked on the loss of politeness by even southern Italians, attributing it, in a lapse of his rich perceptive faculty, to the sense of loneliness and alienism. The cause may just as well, however, be a sense of the trouble of politeness not being worth while.

It may be said that such protests on the part of the younger medical institutions are largely unctuous rectitude. Wait until *you* have got an assured *clientèle*, wait until *you* are run off your legs with looking after them, wait until *you* have experience of their wicked ways! Well, one grants but very little of all this. If one is fortunate in forming one's tradition in a more enlightened day (would be the reply) so much the better, so much the greater margin for the deterioration of the new broom, so much more effective the milder degrees of the authoritarian manner. No, as all things human need a slow, continual reconstruction, so does the British hospital and nursing system; and not least in this present matter of Politeness to Patients. Recent talk about a better designed uniform, all to the good as that is, is of much less importance.

